# Prevalence of Diabetes and Associated Factors in the Uyghur and Han Population in Xinjiang, China

**DOI:** 10.3390/ijerph121012792

**Published:** 2015-10-14

**Authors:** Haiying Gong, Lize Pa, Ke Wang, Hebuli Mu, Fen Dong, Shengjiang Ya, Guodong Xu, Ning Tao, Li Pan, Bin Wang, Guangliang Shan

**Affiliations:** 1 Department of Epidemiology and Statistics, Institute of Basic Medical Sciences, Chinese Academy of Medical Sciences, School of Basic Medicine, Peking Union Medical College, Beijing 100005, China; E-Mails: gonghaiying2802862@163.com (H.G.); wangkehope@126.com (K.W.); fionarab@163.com (F.D.); panli1716@163.com (L.P.); wbpumc@163.com (B.W.); 2 Xinjiang Uyghur Autonomous Region Centre for Disease Control and Prevention, Urumqi 830001, China; E-Mails: 13209963010@163.com (L.P.); kawapqi@sina.com (H.M.); Rzwangul@126.com (S.Y.); 3 Department of Epidemiology and Statistics, College of Public Health, Jilin University, Changchun, 130021, China; E-Mail: xugd13@mails.jlu.edu.cn; 4 Department of Epidemiology and Statistics, College of Public Health, Xinjiang Medical University, Urumqi 830011, China; E-Mail: zflningning@sina.com

**Keywords:** diabetes, associated factors, Uyghur population, Han population

## Abstract

*Objective*: To estimate the prevalence of diabetes and identify risk factors in the Uyghur and Han population in Xinjiang, China. *Methods*: A cross-sectional study in urban and rural areas in Xinjiang, including 2863 members of the Uyghur population and 3060 of the Han population aged 20 to 80 years, was conducted from June 2013 to August 2013. Data on fasting plasma glucose (FPG) and personal history of diabetes were used to estimate the prevalence of diabetes. Data on demographic characteristics, lifestyle risk factors, and lipid profiles were collected to identify risks factors using the multivariate logistic regression model. *Results*: In urban areas, the age- and gender-standardized prevalence of diabetes was 8.21%, and the age- and gender-standardized prevalence of diabetes was higher in the Uyghur population (10.47%) than in the Han population (7.36%). In rural areas, the age- and gender-standardized prevalence of diabetes was 6.08%, and it did not differ significantly between the Uyghur population (5.71%) and the Han population (6.59%). The results of the multivariate logistic regression analysis showed that older age, obesity, high triglycerides (TG), and hypertension were all associated with an increased risk of diabetes in the Uyghur and Han population. Urban residence and low high-density lipoprotein cholesterol (HDL-C) were associated with an increased risk of diabetes in the Uyghur population. Being an ex-drinker was associated with an increased risk of diabetes and heavy physical activity was associated with a decreased risk of diabetes in the Han population. Conclusions: Our study indicates that diabetes is more prevalent in the Uyghur population compared with the Han population in urban areas. Strategies aimed at the prevention of diabetes require ethnic targeting.

## 1. Introduction

Diabetes has been rapidly becoming a global public health problem [1]. The number of patients with diabetes worldwide was estimated at 382 million in 2014 and is expected to rise to 592 million by the year 2035 [2,3]. In China, the prevalence of diabetes rose from 0.9% [4] in 1980 to 11.6% [5] in 2010. Population aging, economic development, urbanization, and lifestyle changes were likely to lead to the growing epidemic of diabetes [6,7].

Xinjiang is located in the northwest of China, where more than 13 different ethnic groups live. In these groups, the Uyghur and Han populations were the largest, comprising approximately 46% and 40% of the total [8], respectively. Information on the prevalence and associated factors of diabetes in these two groups available currently were from studies carried out in those aged over 30 years in limited areas in Xinjiang [8,9,10,11,12]. Thus, we conducted a population-based study in people from the Uyghur and Han populations aged from 20 to 80 years old to update the present data.

## 2. Methods

### 2.1. Participants

This study was based on the data obtained from the China National Health Survey (CNHS). This study adopted a multistage, stratified cluster sampling scheme to select a representative sample of individuals aged 20 to 80 years from June 2013 to August 2013 in the Xinjiang Uyghur Autonomous Region. In the first stage, the whole Xinjiang Uyghur Autonomous Region was stratified into urban and rural areas. Urumqi, Karamay, and Wusu were selected from urban areas and Hami, Toksun, and Wusu were selected from rural areas. In the second stage, four communities (two Uyghur communities and two Han communities) from Urumqi, two Uyghur communities from Karamay, and two Han communities from Wusu; four rural townships (two Uyghur rural townships and two Han rural townships) from Hami, two Uyghur rural townships from Toksun, and two Han rural townships from Wusu were selected. In the third stage, two street districts from every community and two villages from rural townships were selected. Only subjects who were aged 20 to 80 years and had been living in their current residence for one year or longer were eligible to participate (a total of 6684 participants). Of those, a total of 5923 participants (2863 Uyghur and 3060 Han) completed the present study, giving a response rate of 88.6%. This study was approved by the bioethical committee of the Institute of Basic Medical Sciences, the Chinese Academy of Medical Sciences, Beijing, China. Written consent was obtained from all participants before data collection (approval NO. 028-2013).

### 2.2. Data Collection

The questionnaire was administered by trained interviewers who were fluent both in the Uyghur and Han dialogues to obtain information on demographic characteristics, personal medical history, and health behaviors. The interview included questions related to ethnicity, age, gender, location, education level, physical activity, smoking status, drinking status, *etc.* Smoking status was divided into three categories: (1) those who had never smoked (“never smokers”); (2) ex-smokers who consistently had not smoked any cigarettes, cigars, tobacco leaves for more than half a year preceding the survey; (3) current smokers who currently smoke any cigarettes, cigars, or tobacco leaves. Drinking status also was divided into three categories: (1) those who never drank alcohol (“never drinkers”); (2) ex-drinkers who consistently had not drunk any liquor, beer, and fruit wine for more than half a year preceding the survey; (3) current drinkers who currently drink any liquor, beer, and fruit wine. Physical activity was divided into three categories: (1) light: e.g., office worker, salesperson, and house worker; (2) moderate: e.g., driver, electrician, and latheman; (3) heavy: e.g., manual worker, steel worker, and mineworker.

Blood pressure was measured three times consecutively with a one-minute interval between measurements with the participant seated after five minutes of rest, and the mean of three consecutive readings was used for the analyses (Omron HEM-907). Overweight and obesity were assessed by body mass index (BMI) with weight measured on a bioelectrical impendence analysis (BIA) system (Tanita BC-420). Height and weight were measured to the nearest 1.0 cm and 0.1 kg in a standing position, respectively, with participants wearing light clothing. After at least eight hours of overnight fasting, a venous blood sampling was collected using a vacuum tube from each subject at the time of the interview, processed on site within 4 h of collection, and then shipped to the laboratory in Beijing, kept at −80 °C below zero before being analyzed. FPG and lipids, including total cholesterol (TC), triglycerides (TG), high-density lipoprotein cholesterol (HDL-C), and low-density lipoprotein cholesterol (LDL-C), were assessed by Peking Union Medical College Hospital.

### 2.3. Definitions

Diabetes was diagnosed according to the American Diabetes Association (ADA) 2009 criteria: FPG ≥ 7.0 mm/L (126 mg/dL), or/and a previous diagnosis of diabetes. All participants were asked the question “Has a doctor ever told you that you have diabetes?” and “When and where have you been diagnosed with diabetes?” A self-reported previous diagnosis needed to be diagnosed by health care professionals. The presence of hypertension was defined as a systolic blood pressure (SBP) ≥140 mm Hg or/and a diastolic blood pressure (DBP) ≥90 mm Hg, or/and the participant had his/her individual history of hypertension. A BMI of 25–30 kg/m^2^ indicated overweight and a BMI of ≥30 kg/m^2^ was an indicator of obesity [13]. According to ATP III, high TC was defined as a serum level of TC ≥6.21 mmol/L. High TG was defined as a serum level of triglyceride ≥2.26 mmol/L. High LDL-C was defined as a serum level of LDL-C ≥4.16 mmol/L. Low HDL-C was defined as a serum level of HDL-C <1.03 mmol/L. The ratio of TC/HDL-C ≥ 5 was also regarded as abnormal.

### 2.4. Statistical Analysis

Basic characteristics were presented as means ± SD or n (%) according to location in the Uyghur and Han populations. Age- and gender-standardized prevalence of diabetes was calculated by the direct method using population census data of China in 2010. The differences between the two populations were tested by *t*-test or χ^2^ test. Multivariate logistic regression analysis, using a enter method, was performed to estimate the risk factors related to diabetes (diabetes status as a dependent variable; gender, age, BMI, TC, HDL-C, LDL-C, ratio of TC/HDL-C, education level, physical activity, hypertension, smoking status, drinking status as independent variables). The odds ratio (OR) and its 95% confidence interval (CI) were presented to show the risks. *p*-values < 0.05 were considered to be statistically significant. Statistical analysis was performed by SAS software, version 9.3.

## 3. Results

Characteristics associated with diabetes in the Uyghur and Han populations are shown in Table 1. Compared with the Uyghur population living in rural areas, the Uyghur population living in urban areas were more prevalently male, younger, more likely to have a lower TC, TG, SBP, and DBP, and were more likely to smoke and drink (*p* < 0.05). The Han population living in urban areas had a higher TG, LDL-C, and ratio of TC/HDL-C than the Han population living in rural areas (*p* < 0.05). The proportion of people with heavy physical activity was lower in the Han population living in urban areas than in the Han population living in rural areas (*p* < 0.05).

**Figure 1:**
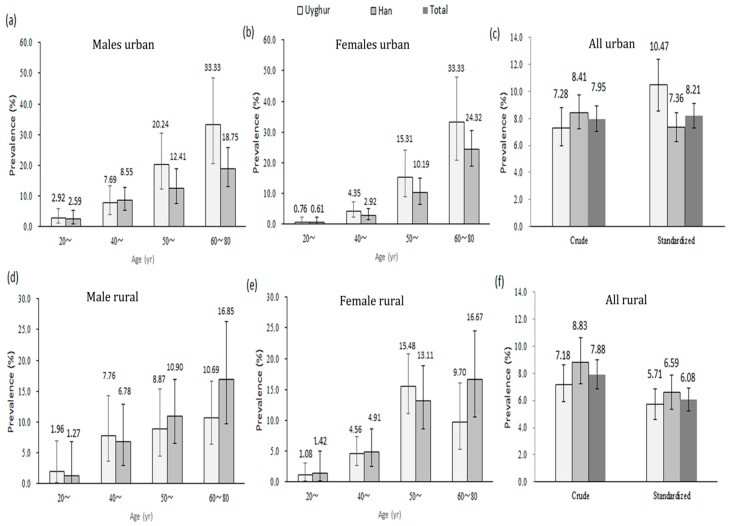
Age-specific prevalence of diabetes in Uyghur and Han populations. (**a**) The age-specific prevalence of diabetes in males in Uyghur and Han populations in urban areas; (**b**) The age-specific prevalence of diabetes in females in Uyghur and Han populations in urban areas; (**c**) The crude and age- and gender-standardized prevalence of diabetes in Uyghur and Han populations in urban areas; (**d**) The age-specific prevalence of diabetes in males in Uyghur and Han populations in rural areas; (**e**) The age-specific prevalence of diabetes in females in Uyghur and Han populations in rural areas; (**f**) The crude and age- and gender-standardized prevalence of diabetes in Uyghur and Han populations in rural areas.

Figure 1 shows the age-specific prevalence of diabetes in the Uyghur and Han populations. In urban areas, the crude prevalence of diabetes was 7.95% (7.28% for the Uyghur population and 8.41% for the Han population, respectively). The age- and gender-standardized prevalence of diabetes was 8.21%, and it was significantly higher in the Uyghur population than in the Han population (10.47% *vs.* 7.36%, *p* < 0.05). The age-specific prevalence of diabetes increased significantly with increased age in the Uyghur and Han populations in both genders (*p* < 0.05). There was no significant difference in the age-specific prevalence of diabetes between the Uyghur and Han populations except for those aged 60~80 years (33.33% *vs.* 18.75%, *p* < 0.05).

In rural areas, the crude prevalence of diabetes was 7.88% (7.18% for the Uyghur population and 8.83% for the Han population, respectively). The age- and gender-standardized prevalence of diabetes was 6.08%, and it did not differ significantly between the Uyghur and Han populations (5.71% *vs.* 6.59%, *p* > 0.05). The age-specific prevalence of diabetes increased significantly with increased age in the Uyghur and Han populations in both genders (*p* < 0.05). There was no significant difference in the age-specific prevalence of diabetes between the Uyghur and Han populations in both genders (*p* > 0.05).

**Table 1:** Characteristics of Uyghur and Han populations according to location.

Characteristics	Uyghur (*n* = 2863)		Han (*n* = 3060)	
	Urban (*n* = 1359)	Rural (*n* = 1504)	Urban (*n* = 1950)	Rural (*n* = 1110)
Male (%)	515 (37.90)	501 (33.31) *	809 (41.49) **^†^**	442 (39.82) **^‡^**
Age, years	41.03 ± 11.95	48.69 ± 12.33 *	47.38 ± 13.74 **^†^**	49.73 ± 11.20 ** ^‡^
20~	636 (48.80)	381 (25.33) *	596 (30.56) **^†^**	220 (19.82) ** ^‡^
40~	442 (32.52)	467 (31.05)	611 (31.33)	342 (30.81)
50~	182 (13.39)	363 (24.14)	361 (18.51)	339 (30.54)
60~80	99 (7.28)	293 (19.48)	382 (19.59)	209 (18.83)
BMI, kg/m^2^	25.87 ± 4.73	25.72 ± 4.22	24.56 ± 3.66 **^†^**	24.66 ± 3.64 **^‡^**
<25.0	607 (44.80)	661 (44.45)	1114 (57.30) **^†^**	614 (55.67) **^‡^**
25.0~	517 (38.15)	613 (41.22)	679 (34.93)	404 (36.63)
30.0~	231 (17.05)	213 (14.32)	151 (7.77)	85 (7.71)
TC (mmol/L)	4.74 ± 1.00	4.84 ± 1.07 *	4.83 ± 1.01 **^†^**	4.65 ± 0.96 ** ^‡^
<6.21	1248 (91.90%)	1372 (91.65%)	1775 (91.07%)	1043 (94.22%) ** ^‡^
≥6.21	110 (8.10%)	125 (8.35%)	174 (8.93%)	64 (5.78%)
TG (mmol/L)	1.46 ± 1.28	1.74 ± 1.61 *	1.75 ± 1.70 **^†^**	1.66 ± 1.85
<2.26	1148 (84.60%)	1161 (77.71%) *	1531 (78.55%) **^†^**	915 (82.73%) ** ^‡^
≥2.26	209 (15.40%)	333 (22.29%)	418 (21.45%)	191 (17.27%)
HDL-C (mmol/L)	1.36 ± 0.26	1.37 ± 0.27	1.44 ± 0.31 **^†^**	1.45 ± 0.30 **^‡^**
<1.03	132 (9.72%)	135 (9.02%)	135 (6.93%) **^†^**	73 (6.59%) **^‡^**
≥1.03	1226 (90.28%)	1362 (90.98%)	1814 (93.07%)	1034 (93.41%)
LDL-C (mmol/L)	2.90 ± 0.82	2.92 ± 0.79	2.81 ± 0.79 **^†^**	2.68 ± 0.74 ** ^‡^
<4.16	1267 (93.30%)	1412 (94.32%)	1848 (94.82%)	1074 (97.02%) ** ^‡^
≥4.16	91(6.70%)	85 (5.68%)	101 (5.18%)	33 (2.98%)
Ratio of TC/HDL-C	3.60 ± 0.96	3.65 ± 1.02	3.46 ± 0.91 **^†^**	3.31 ± 0.84 ** ^‡^
<5.00	1247 (91.83%)	1384 (92.45%)	1864 (95.64%)	1078 (97.38%)
≥5.00	111 (8.17%)	113 (7.55%)	85 (4.36%)	29 (2.62%)
Glucose level	5.31 ± 1.62	5.08 ± 1.55 *	5.19 ± 1.44 **^†^**	5.33 ± 1.46 ** ^‡^
Education level (%)				
Illiteracy and primary school	139 (10.29)	576 (38.61) *	236 (12.13) **^†^**	409 (36.91) ** ^‡^
Junior and senior high school	594 (43.97)	745 (49.93)	920 (47.28)	640 (57.76)
Graduate or above	618 (45.74)	171 (11.46)	790 (40.60)	59 (5.32)
Physical activity (%)				
Light	1028 (75.81)	549 (36.65) *	1602 (82.15) **^†^**	312 (28.11) ** ^‡^
Moderate	279 (20.58)	199 (13.28)	276 (14.15)	134 (12.07)
Heavy	49 (3.61)	750 (50.07)	72 (3.69)	664 (59.82)
SBP (mm Hg)	116.80 ± 16.70	122.10 ± 18.33 *	119.40 ± 16.46 **^†^**	121.40 ± 17.08 **
DBP (mmHg)	71.93 ± 11.29	73.70 ± 11.96 *	74.16 ± 10.95 **^†^**	73.67 ± 10.98
Smoking Status (%)				
Never smokers	973 (71.65)	1169 (77.88) *	1353 (69.49) **^†^**	752 (67.75) **^‡^**
Ex-smokers	85 (6.26)	105 (7.00)	189 (9.71)	110 (9.91)
Current smokers	300 (22.09)	227 (15.12)	405 (20.80)	248 (22.34)
Drinking Status (%)				
Never drinkers	1017 (75.11)	1232 (82.30) *	1000 (51.68) **^†^**	572 (51.91) **^‡^**
Ex-drinkers	113 (8.35)	144 (9.62)	101 (5.22)	76 (6.90)
Current drinkers	224 (16.54)	121 (8.08)	834 (43.10)	454 (41.20)

Data are presented as means ± SD or *n* (%). * denotes *p* < 0.05 for the difference between urban and rural residents in the Uyghur population; ** denotes *p* < 0.05 for the difference between urban and rural residents in the Han population; **^†^** denotes *p* < 0.05 for the difference between the Uyghur and Han populations in urban areas; **^‡^** denotes *p* < 0.05 for the difference between the Uyghur and Han populations in rural areas.

**Figure 2:**
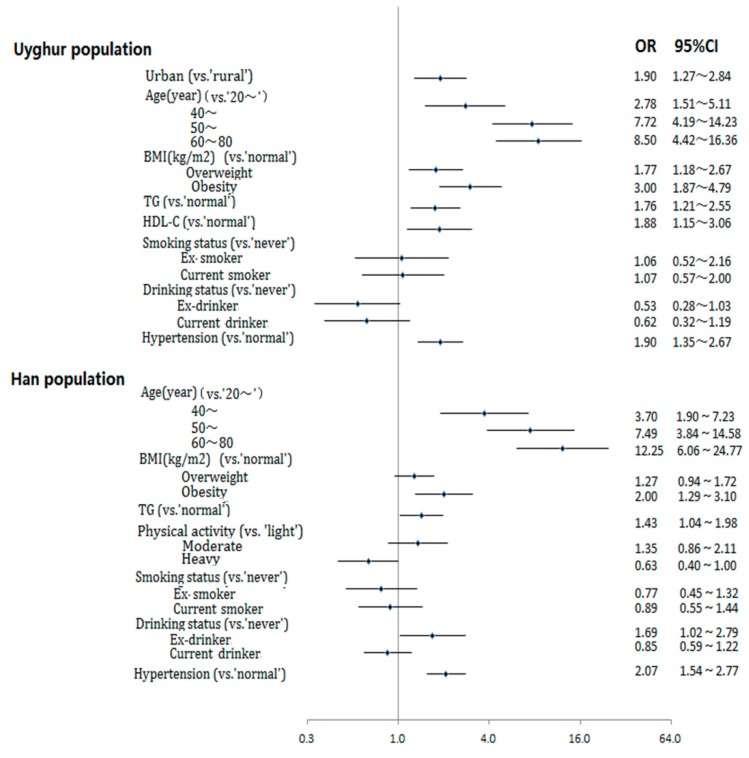
Multivariate logistic regression analysis of risk factors of diabetes in Uyghur and Han populations.

Figure 2 shows multivariate logistic regression analysis of risk factors of diabetes in the Uyghur and Han populations. In the Uyghur population, multivariate logistic regression analysis revealed that urban residence, older age, overweight, obesity, high TG, low HDL-C, and hypertension were all associated with an increased risk of diabetes (*p* < 0.05). In the Han population, multivariate logistic regression analysis revealed that older age, obesity, high TG, ex-drinkers, and hypertension were all associated with an increased risk of diabetes (*p* < 0.05), and heavy physical activity was associated with a decreased risk of diabetes (*p* < 0.05). No association of diabetes was observed with regard to smoking status in the Uyghur and Han populations (*p* > 0.05).

## 4. Discussion

Our study found that the age- and gender-standardized prevalence of diabetes was 8.21% (10.47% for the Uyghur population and 7.36% for the Han population) in urban areas and was 6.08% (5.71% for the Uyghur population and 6.59% for the Han population) in rural areas, respectively. Diabetes was more prevalent in the Uyghur population compared with the Han population in urban areas. The differences in the prevalence of diabetes might be due to differences in dietary habit. The Uyghur populations were more likely to have a dietary pattern involving the consumption of a lot of beef, mutton, and butter, which lead to the possibility of developing diabetes. In rural areas, due to the less developed economic conditions, especially in some regions inhabited by the Uyghur population, the reduction in the beef and butter of the diet in the Uyghur population might well account for the decreased risk of diabetes. It was therefore not difficult to understand the similar prevalence of diabetes between the Uyghur and Han populations in rural areas.

The prevalence of diabetes in our finding was different from earlier reports in Xinjiang. Tao *et al.* investigated 1571 adults from the Uyghur population aged 20 to 80 years from 2006 to 2007, and the results showed that the age- and gender-standardized prevalence of diabetes was 8.16% [14]. Tao *et al.* reported that the age-standardized prevalence of diabetes was 5.4% in the Uyghur population and 7.1% [15] in the Han population among 4695 adults from the Uyghur population and 3717 adults from the Han population aged 35 to 74 years in seven cities in Xinjiang from 2007 to 2010. Awuti *et al.* investigated 1043 adults from the Uyghur population aged >20 years in the town of Cele in the Xinjiang Hetian region in 2010, and the results showed that the prevalence of diabetes was 9.0% [11]. Overall, methodological differences in sampling and the differences in the study population might contribute to the differences in the prevalence of those studies. The prevalence of diabetes in the study was lower than the level in Xinjiang in the 2010 China Noncommunicable Disease Surveillance report (14.1%) [5], and also lower than the level in a nationally representative sample of Chinese adults (11.6%) [5]. This difference might be explained by different diagnostic criteria for diabetes; the diagnosis of diabetes in the study was established on the basis of FPG, which had a lower sensitivity relative to oral glucose tolerance test (OGTT) diagnosis [16], and, therefore, the prevalence of diabetes might have been underestimated in the study [17].

The positive association between age and diabetes has been previously observed in the Uyghur population [11] in Xinjiang and in other minorities such as the Kazakh [12], Manchu, and Koreans [18] in China, which was consistent with our results. As a widely used index for measuring obesity, BMI was used to predict diabetes for many years. Additionally, waist circumference, waist-to-hip ratio and waist-to height were also used for the detection of diabetes. Currently, there were some debates about which anthropometric parameters were the best predictors of the risk of diabetes, but the superiority of these measures was still controversial [19,20,21]. In the epidemiological survey, it was well established that the reliability of weight and height measurements was higher than the reliability of waist circumference and hip circumference measurements [22]; thus, in our study, BMI was used to measure the degree of obesity to predict diabetes.

Several previous studies have documented that high TG and low HDL-C were found to be risk factors for diabetes [23,24]. High TG could be used as a unique diagnostic marker for insulin resistance and other cardiovascular diseases [25]. HDL-C might have the ability to stimulate the release of insulin [26], which reduces the possibility of developing diabetes [27]. However, the association between HDL-C and diabetes was not statistically significant in the Han population. It suggested that screening for diabetes might not be efficient if we focused on HDL-C in the Han population. As observed in the other population [6,28], the risk of diabetes decreased as physical activity increased in the Han population, suggesting that lifestyle modification, such as increased physical activity, might contribute to the prevention of diabetes in the Han population.

A large prospective study showed that the incidence of diabetes was 2.49 times higher in ex-drinkers than in the people who never drank and were also male [29]. In the present study, our results showed that the risk was 1.69 times higher in ex-drinkers than in those who never drank in the Han population. Subsequently, we separately estimated the relationship between drinking status and diabetes in males and females. The results showed that the risk in male ex-drinkers was 2.03 more than in those who never drank, and no association was found between drinking status and diabetes in females. People who excessively drank were more likely to suffer from diabetes, and in order to prevent the progress of diabetes, drinkers with diabetes abstained from wine. Therefore, it was not difficult to understand the higher risk of diabetes in ex-drinkers.

There were several potential limitations in our study. First, our study was cross-sectionally designed, which made it hard to draw conclusions about the causal relationships. Second, a dietary survey was not included in our study, but the reliability of a dietary survey was not easily warranted in large epidemiological research. Third, the current study only paid attention to environmental factors. Further genetic studies should be carried out. Fourth, we did not differentiate type 1 from type 2 diabetes in the study.

## 5. Conclusions

Our study indicates that diabetes is more prevalent in the Uyghur population compared with the Han population in urban areas. Strategies aimed at the prevention of diabetes require ethnic targeting.
